# Gene detection in a family with monilethrix and treatment with 5% topical minoxidil

**DOI:** 10.1111/srt.13233

**Published:** 2022-11-16

**Authors:** Qingli Shen, Zhonghua Fu, Pengqiang Du, Jianbo Wang

**Affiliations:** ^1^ Department of Pharmacy Henan Provincial People's Hospital Zhengzhou University People's Hospital Henan University People's Hospital Zhengzhou Henan China; ^2^ Department of Pharmacy Henan Provincial People's Hospital Fuwai Central China Cardiovascular Hospital Central China Fuwai Hospital of Zhengzhou University Zhengzhou Henan China; ^3^ Department of Dermatology Henan Provincial People's Hospital Zhengzhou University People's Hospital Henan University People's Hospital Zhengzhou Henan China

**Keywords:** 5% minoxidil, *KRT86* gene, monilethrix

## Abstract

**Objective:**

To determine the causative gene mutation in a family with monilethrix and observe the therapeutic effect of 5% topical minoxidil.

**Method:**

Clinical data from a family with monilethrix were collected. Peripheral blood samples were taken from the proband, the parents, and 100 unrelated healthy controls. Genomic DNA was extracted. The genetic variation sites were screened with exome sequencing and verified by Sanger sequencing. The proband was treated with 5% topical minoxidil (1 mL twice daily). Hair quality was examined by dermoscopy before and after treatment.

**Results:**

The proband and her father have the heterozygous missense variant c.1204G > A (p.E402K) in exon 7 of the *KRT86* gene. However, the mutation was not found in the mother and healthy controls. The proband was treated with 5% topical minoxidil. Hair density and hair shaft quality improved significantly after 6 months of treatment. No adverse events occurred during treatment.

**Conclusion:**

This study shows that p.E402K is a mutation “hot spot” in patients with autosomal dominant monilethrix in China. Treatment with 5% topical minoxidil, is safe and effective.

## 1 INTRODUCTION

Monilethrix (OMIM 158000) is a rare congenital dystrophic hair loss. It is characterized by periodic beaded hair shafts, sparse hair, hair fragility, and follicular keratosis. The hair shafts show elliptical nodes separated by abnormally narrow intermodal segments. There are two modes of inheritance: autosomal dominant and autosomal recessive.^1^, ^2^ There is no cure for monilethrix. Reduction in hairdressing trauma may diminish weathering and improve severely affected cases.^3^ Topical or oral minoxidil has been used to treat monilethrix and noted a reduction in fragility and increased hair length after a few months of treatment.^3^, ^4^

Here, we reported a patient with an autosomal dominant monilethrix. The gene mutation site was identified by exome sequencing and verified by Sanger sequencing. The therapeutic effect of topical minoxidil (5% solution) for monilethrix was observed.

## 2 MATERIALS AND METHODS

### 2.1 Ethics approval

This study was approved by the Ethics Committee of Henan Provincial People's Hospital (ethics approval No: 2019‐07). Written consent was obtained from study subjects.

### 2.2 Blood sample collection and DNA extraction

Blood samples were obtained from the patient and the parents. Whole blood genomic DNA was extracted with the TIANamp whole blood genomic DNA extraction kit (Tiangen Biochemical Technology [Beijing] Co., Ltd.). The same method was used to extract genomic DNA from 100 unrelated healthy individuals as a control.

### 2.3 Exome library construction and sequencing

Customized probes, based on the known causative genes in the OMIM database as of May 2020, were obtained from Shanghai WeHealth BioMedical Technology Co., Ltd. The entire exome library was captured after TWIST synthesis, followed by the construction of the full exome library of the proband. The procedures were briefly described as follows. The hybridization reaction system was configured according to the kit instruction, and the reaction was performed on the polymerase chain reaction (PCR) machine. Overnight hybridization: The capture library was obtained by configuring the capture reaction system according to the kit instructions. The amplification reaction system was configured and placed on the PCR instrument for reaction. The PCR reaction program was (1) 98°C 45 s, 98°C 15 s, 60°C 30 s, and 72°C 30 s, cycling for six times, (2) 72°C 5 min, and (3) hold at 4°C. The product was quantified after magnetic bead purification and stored at −20°C. Paired‐end sequencing was performed on the MGISEQ‐T7 sequencing platform using PE100 mode. The identified variant loci were sequenced by Sanger sequencing. The loci were verified in the proband, the parents, and the healthy controls. All experiments were performed by Shanghai WeHealth BioMedical Technology Co., Ltd.

## 3 RESULTS

### 3.1 Clinical information collection

**FIGURE 1:**
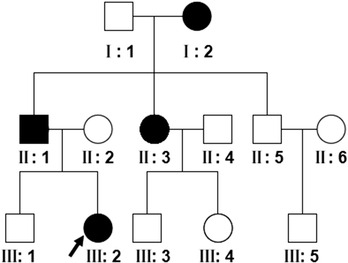
The pedigree structure of the family (arrow, the proband)

The proband (III‐2) was a 16‐year‐old girl of nonrelated parents who presented with sparse and brittle hair. She was born with full hair. Her hair gradually became brittle, fragile, dry, and lusterless around 2 months old. The hair progressively became thinned, especially on the scalp and the occipital area. The condition was aggravated in winter but relieved in spring without subjective symptoms. The proband's father, aunt, and grandmother had similar symptoms (Figure 1).

**FIGURE 2:**
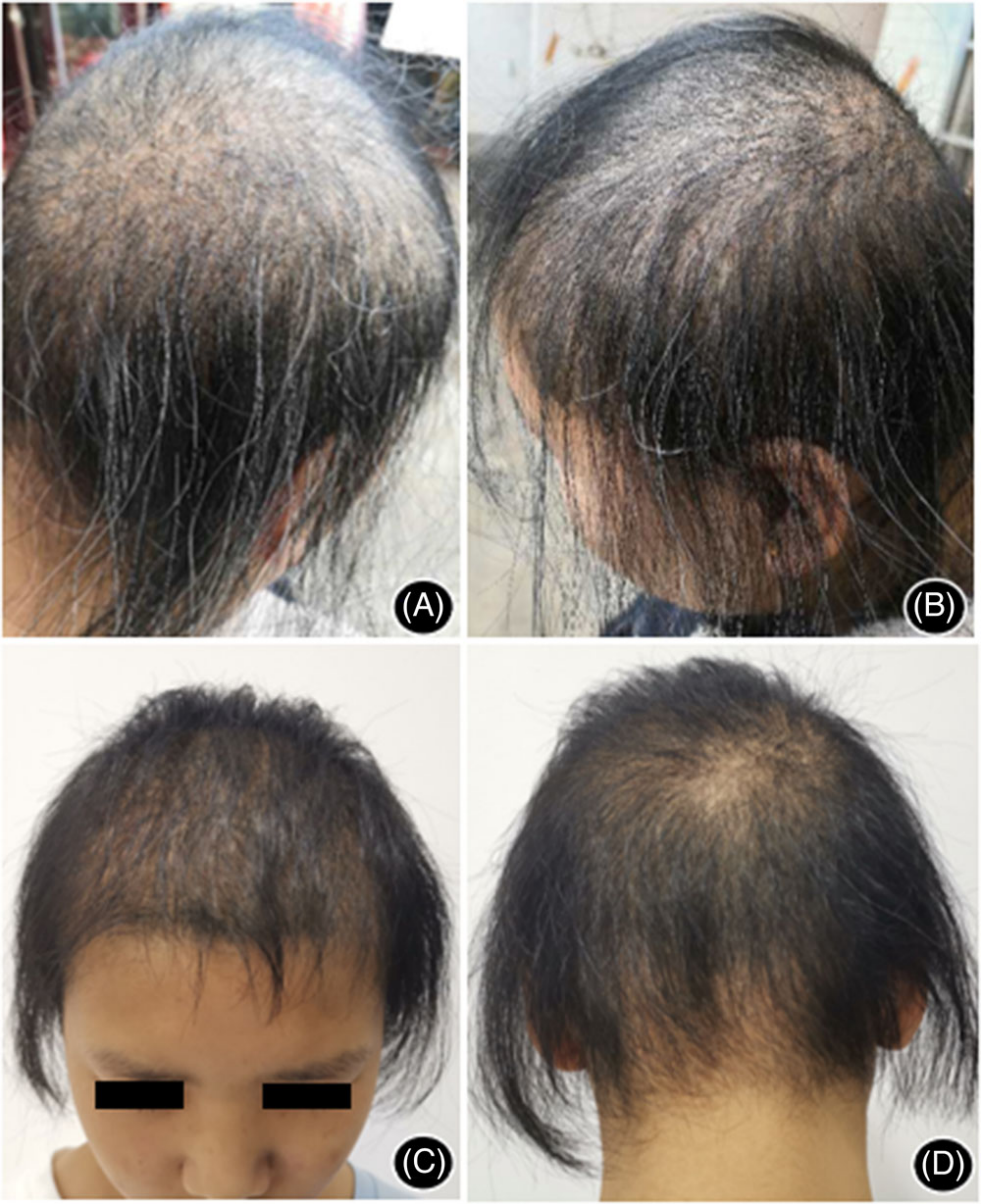
The clinical manifestations of the proband before and after treatment. The proband had sparse and dry hair, mainly on the scalp and the occipital area (A and B). After treatment, hair density increased and hair shaft became shiny (C and D).

The patient was healthy with average intelligence. There were no abnormalities in the ears, teeth, and nails. Examination found sparse hair with an exposed scalp, especially on the top of the head and the occipital area. Most of the hair attached to the scalp was about 2 cm long. Fractured hair and abnormal hair shafts were noted. Visual inspection showed multiple beaded nodules with white hair among them (Figure 2A,B). No abnormalities were observed in the eyebrows, eyelashes, and fingernails.

### 3.2 Pre‐ and posttreatment

**FIGURE 3:**
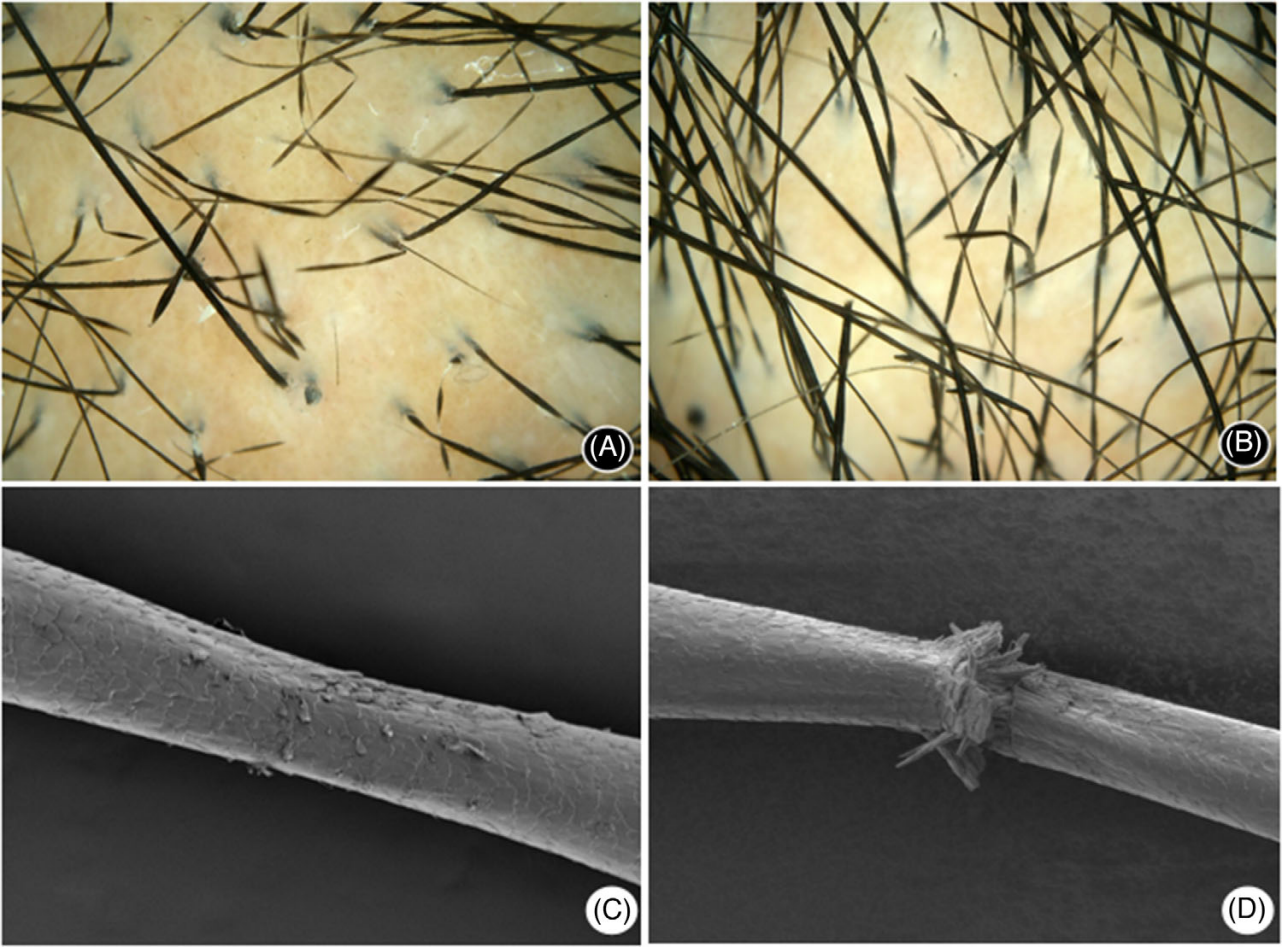
Proband's dermoscopy and scanning electron microscope (SEM) images. (A) Before treatment, dermatoscopy (50×) shows that swelling and narrowing of the legions occurred alternately, and some legions broke at constrictions. (B) After treatment, hair density increased, and fewer abnormal hairs were observed (50×). (C) The appearance of monilethrix under SEM (3 kV 11.2 mm × 300). (D) Fractured hair under SEM (3 kV 11.2 mm × 300)

Dermoscopy (50×) found alternately swelled and narrowed dystrophic hair with typical beaded hair shafts. Some hair were broken at constrictions (Figure 3A). Scanning electron microscopy showed largely normal imbricate hair cuticles in the swelled dystrophic hair. Many cuticles were destroyed, broken, and disappeared in the narrowed lesions with apparent longitudinal ridges and grooves (Figure 3C). The hair shaft was not entirely broken at constrictions, and the cross section was irregular and staggered (Figure 3D).

The patient was diagnosed with autosomal dominant monilethrix. Treatment started with topical minoxidil (5% solution), spraying six‐to‐seven times (1 mL) on the entire head, once in the morning and once in the evening. The drug was evenly spread on the scalp and massaged for 1–2 min to promote absorption. The patient found a significant decrease in hair loss after 2 months. After 6 months of treatment, hair density and quality improved significantly. The hair shaft was slightly shiny with reduced hair breakage, and the white hair disappeared. The dermoscopy (50×) showed that the hair density (in the same position before treatment) increased. Although the monilethrix and hair breakage at constrictions were still present, the amount was reduced (Figure 3B). The patient continued treatment for another 6 months. Initial hair density and quality improvement were maintained without further improvement. No adverse events were observed during minoxidil treatment.

### 3.3 Gene monitoring results

**FIGURE 4:**
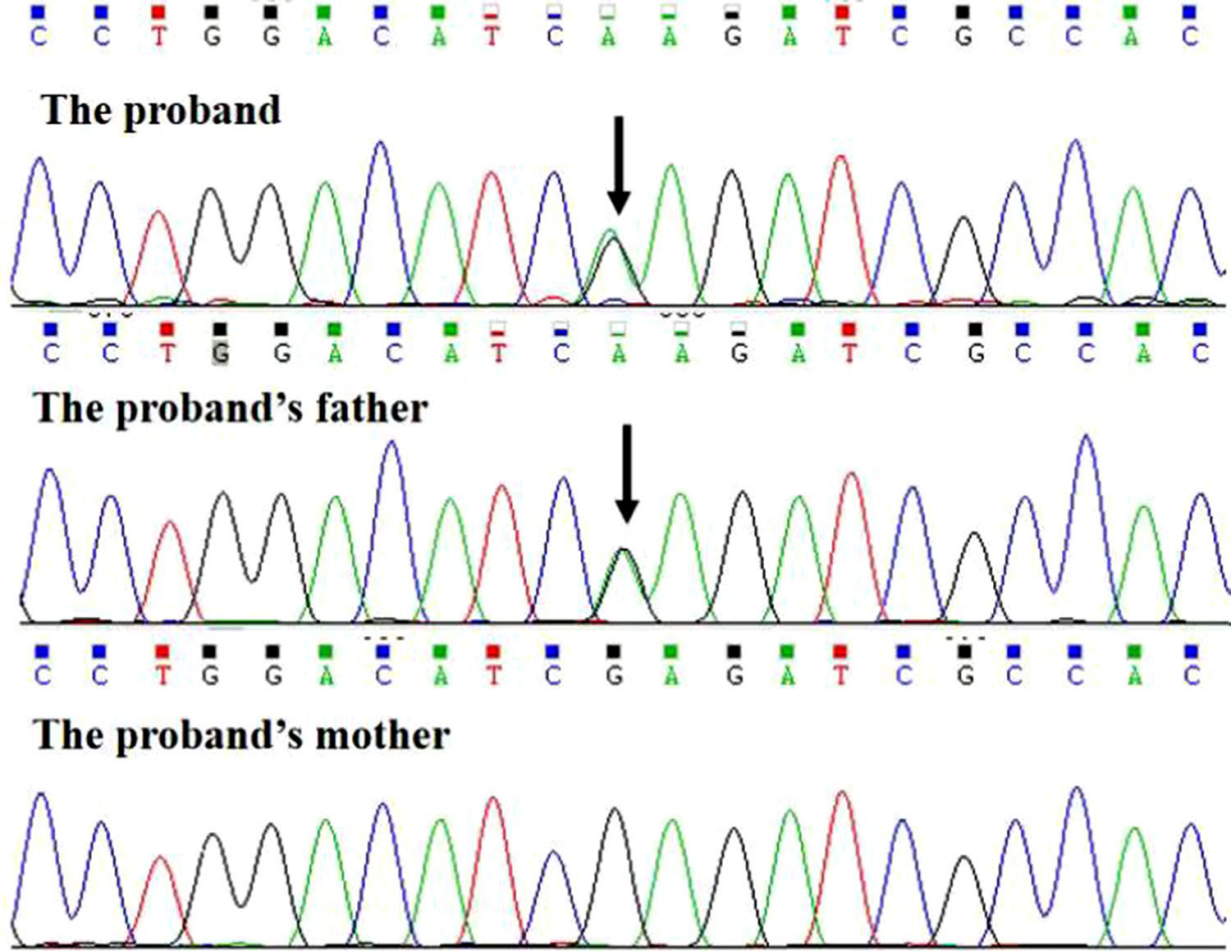
Partial sequencing results of *KRT86* gene of the proband and the parents. The arrows show the locus of variation.

The proband and her father have a heterozygous missense variant c.1204G > A in exon 7 of the *KRT86* gene. The amino acid at position 402 of one of the proteins encoded by the *KRT86* gene changed from glutamic acid to lysine (p.E402k). This variant was not found in the mother of the proband or the healthy controls (Figure 4).

## 4 DISCUSSION

The mutation sites responsible for autosomal dominant monilethrix are located in the *KRT81*/*hHb1*, *K* *R* *T* *83*/*hHb3*, and *KRT86*/*hHb6* genes,^1^, ^5^, ^6^ which encode the human type II hair keratin. Mutations of the gene loci in the highly conserved regions of these genes causes changes in the base sequence. The changes affect the arrangement of amino acids and the keratin conformation, leading to the destruction of the normal structure or function of the protein. In this family, a heterozygous missense variant c.1204G > A is present in exon 7 of the *KRT86* gene. This causes the amino acid at position 402 of one of the encoded proteins to change from glutamic acid to lysine. The mutation can cause a conformational change in the peptide chain in the corresponding region and a change in the electrostatic interaction force between atoms and groups, thereby affecting the assembly and stability of the intermediate filament of the type II hair keratin.^7^ Because it is a heterozygous variant, only half of the encoded protein is affected. The patient's hair appears to be enlarged and narrowed evenly and alternately in a beaded pattern. Under the scanning electron microscope, the hair cuticles of the enlarged part were essentially normal and arranged in imbricates. However, many hair cuticles in the narrowed part were destroyed, broken, and disappeared. Therefore, the patient's hair was dry, lusterless, and easy to break at constrictions.

Many autosomal dominant monilethrix pedigrees have been reported worldwide. Sixteen mutation sites have been found in gene mutation research, primarily in the *KRT86*/*hHb6* gene (p.E402K, p.E402Q, p.R408P, p.L409P, p.L410P, p.E413D, p.E413K, p.N114D, p.N114H, p.A118E, p.R430Q, and p.V260‐L263del). In contrast, there are only four mutation sites in *KRT81*/*hHb1* (p.E402K and p.E413K) and *KRT83*/*hHb3* genes (p.E407K and p.E418K), respectively. Studies published by Chinese researchers reported six mutation sites in the *KRT86*/*hHb6* gene (p.E402K, p.R408P, p.E413K, p.R430Q, and p.V260‐L263del) and one in the *KRT81*/*hHb1* gene (p.G52R).^8^, ^9^, ^10^, ^11^ Furthermore, reports from outside China suggest that p.E413K in the *KRT86*/*hHb6* gene is a “hot spot” mutation site in patients with autosomal dominant inheritance. In contrast, the hot spot variant detected in China is p.E402K. The gene mutation of the patients in this family is also p.E402K. At present, by searching the domestic and foreign literature, 21 Chinese patients with moniliform hair were reported with genetic test results, and 13 cases had mutation locus p.E402K, accounting for 61.9%.^8^, ^9^, ^11^ Therefore, the causative genes in patients with autosomal dominant monilethrix are ethnically different.

Currently, there is no effective treatment for the disease. It is best to avoid external damage during washing and combing due to the increased brittleness of the patient's hair. Acitretin (0.5 mg/kg/d) oral administration improves follicular keratotic papules in animal experiments and clinical studies. However, there is no apparent effect on the structure and fragility of the hair shaft.^12^ Minoxidil has a specific therapeutic effect on various hair diseases. Several studies have shown that oral or topical minoxidil could effectively treat monilethrix.^3^, ^4^ In this study, the proband was treated with topical minoxidil. Hair loss was improved at 2 months with a significantly improved hair density and hair shaft quality at 6 months, consistent with previous reports. The mechanisms of minoxidil to improve monilethrix are perhaps (1) by stimulating epithelial cell proliferation and differentiation in the hair follicle, (2) by promoting angiogenesis and increasing local blood supply, and (3) by opening potassium channels, preventing calcium ions from flowing into cells, and alleviating the inhibition of epidermal growth factor in hair growth.^3^, ^4^

In conclusion, in this study, a missense variant of the *KRT86* gene c.1204G > A (p.E402K) was detected in a Han family with autosomal dominant monilethrix. It is further proof that p.E402K is a hot spot variant in Chinese patients with monilethrix. At the same time, minoxidil topical treatment was safe and effective. These findings can provide a reference for clinicians in diagnosing and treating monilethrix, genetic counseling, and prenatal diagnosis.

## CONFLICT OF INTEREST

Authors declare no conflict of interest.

## ETHICS STATEMENT

This study was approved by the Ethics Committee of Henan Provincial People's Hospital (ethics approval No: 2019‐07). Written consent was obtained from study subjects.

## Data Availability

Data sharing is not applicable to this article as no new data were created or analyzed in this study.
